# Impaired haematopoietic stem cell differentiation and enhanced skewing towards myeloid progenitors in aged caspase-2-deficient mice

**DOI:** 10.1038/cddis.2016.406

**Published:** 2016-12-01

**Authors:** Swati Dawar, Nur Hezrin Shahrin, Nikolina Sladojevic, Richard J D'Andrea, Loretta Dorstyn, Devendra K Hiwase, Sharad Kumar

**Affiliations:** 1 Centre for Cancer Biology, University of South Australia, Adelaide, South Australia 5001, Australia; 2 Department of Haematology, SA Pathology, Adelaide, South Australia 5000, Australia

## Abstract

The apoptotic cysteine protease caspase-2 has been shown to suppress tumourigenesis in mice and its reduced expression correlates with poor prognosis in some human malignancies. Caspase-2-deficient mice develop normally but show ageing-related traits and, when challenged by oncogenic stimuli or certain stress, show enhanced tumour development, often accompanied by extensive aneuploidy. As stem cells are susceptible to acquiring age-related functional defects because of their self-renewal and proliferative capacity, we examined whether loss of caspase-2 promotes such defects with age. Using young and aged *Casp2*^−/−^ mice, we demonstrate that deficiency of caspase-2 results in enhanced aneuploidy and DNA damage in bone marrow (BM) cells with ageing. Furthermore, we demonstrate for the first time that caspase-2 loss results in significant increase in immunophenotypically defined short-term haematopoietic stem cells (HSCs) and multipotent progenitors fractions in BM with a skewed differentiation towards myeloid progenitors with ageing. Caspase-2 deficiency leads to enhanced granulocyte macrophage and erythroid progenitors in aged mice. Colony-forming assays and long-term culture-initiating assay further recapitulated these results. Our results provide the first evidence of caspase-2 in regulating HSC and progenitor differentiation, as well as aneuploidy, *in vivo*.

Caspase-2, a CARD-containing caspase, is the most evolutionarily conserved member of the caspase family.^1, 2, 3^ In addition to its function in cell death, it has been shown to act as a tumour suppressor (reviewed in Puccini *et al.*^4^). For instance, *CASP2* on human Ch7q is frequently deleted in haematological malignancies^5^ and reduced *CASP2* expression is noted in Burkitt's lymphoma, mantle cell lymphoma, chronic lymphocytic leukaemia (CLL) and hairy cell leukaemia, and correlates with poor prognosis in acute myeloid leukaemia (AML) and acute lymphocytic leukemia (ALL).^6, 7, 8^ In The Cancer Genome Atlas (TCGA) (http://cancergenome.nih.gov/) and BloodSpot databases, lower *CASP2* expression is clearly linked to poor patient survival in AML (http://servers.binf.ku.dk/bloodspot/?gene=CASP2&dataset=normal_human_v2_with_AMLs).

The correlative observations indicating a role for caspase-2 in human malignancies are supported by experimental evidence in mouse models of tumours. For example, caspase-2 deficiency enhances lymphomagenesis in *Eμ-Myc* transgenic mice that develop B-cell lymphoma^9, 10^ and in *ataxia telangiectasia mutated (Atm)-*deficient mice that spontaneusly develop thymic lymphoma.^11^ Furthermore, *MMTV/c-neu*-driven mammary carcinoma,^12^ K-Ras-driven lung carcinoma^13^ and diethylnitrosamine-mediated hepatocellular carcinoma^14^ also show more rapid develoment of tumours in *caspase-2*-deficient (*Casp2*^−/−^) mice. In addition, *in vitro* studies demonstrate that mouse embryonic flibroblasts (MEFs) derived from *Casp2*^−/−^ mice become immortalized more readily and show enhanced sensitivity to transformation by oncogenes, including Ras and cMyc.^9, 15^ Interestingly though, not all types of tumours are affected by the loss of caspase-2,^10, 16^ suggesting that the nature of cell types involved (such as their proliferative capacity) may determine the participation of caspase-2 as a tumour suppressor. One commonly observed feature of *caspase-2*-deficient tumours and MEFs is enhanced aneuploidy,^4, 11, 12, 14, 15, 17^ likely because of reduced or inefficient apoptotic removal of aberrant cells in the absence of caspase-2.

In addition to enhanced sensitivity to tumourigenesis, *Casp2*^−/−^ mice show signs of premature ageing including increased oxidative stress and DNA damage.^18, 19^ Our previous work suggests that the ageing phenotype is partly because of increased oxidative stress-induced damage and impaired antioxidant response.^18, 20^ In oxidative challenge experiments we observed increased tissue damage, accompanied by higher serum IL-6 and IL-1*β* levels in *Casp2*^−/−^ mice compared with wild-type (WT) animals.^14, 20^ More recent studies suggest a role for caspase-2 in the regulation of age-related proteostasis, energy metabolism, lipid metabolism and sex-specific alterations in glucose homeostasis.^21, 22^ As loss of caspase-2 increases genomic instability and predisposes mice to various types of tumours we hypothesize that caspase-2 deficiency results in a compromised state that is more susceptible to various oxidative and oncogenic insults, including that associated with ageing.

Stem cells are highly susceptible to acquiring age-related phenotypic and functional changes, such as aneuploidy, DNA damage and skewed differentiation potential, owing to their high self-renewal and regenerative capacities.^23, 24^ Therefore, this study examined whether caspase-2 deficiency could exacerbate these age-related changes in the haematopoietic stem cell (HSC) and haematopoietic stem and progenitor cell (HSPC) lineages in the bone marrow (BM). Our data suggest that aged *Casp2*^−/−^ mice indeed show significant impairment of HSC differentiation and enhanced skewing towards myeloid progenitors compared with WT mice. Furthermore, our data also suggest that caspase-2 deficiency alone is sufficient for increased number of aneuploid cells in the BM of aged animals.

## Results

The *CASP2* data in BloodSpot suggest high-level expression in the myeloid lineage including common myeloid precursor cells, granulocyte monocyte progenitors, early and late promyelocytes, myelocytes and megakaryocyte erythoid progenitor cells. This observation, combined with a putative role for caspase-2 in haematopoietic tumours, suggested that caspase-2 function may be of particular significance in the haematopoietic lineage.

**Figure 1:**
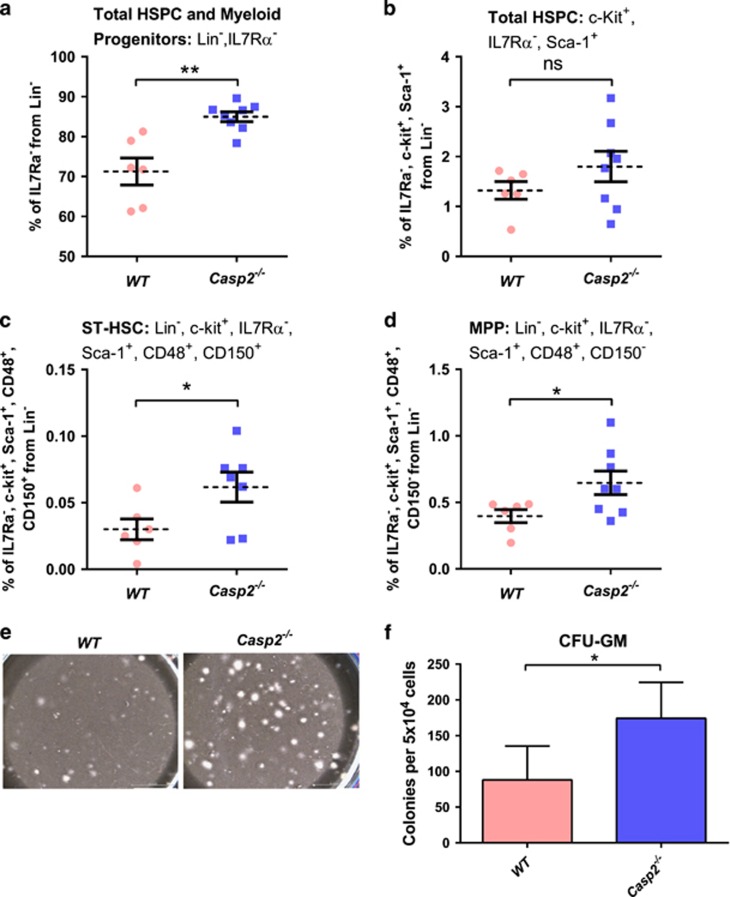
Functional characterization of HSPCs in *Casp2*^−/−^ mice. (**a**) HSPC and myeloid progenitor cells from Lin^−^, IL7R*α*^−^ bone marrow cells quantitated by flow cytometry. Results were obtained from WT (*n*=6) and *Casp2*^−/−^ (*n*=8) mice at 24 months. Data represented as mean±S.E.M., **P<0.01. (**b**) The percentage of total HSPC cells from c-Kit^+^, IL7R*α*^−^, Sca-1^+^ population in the bone marrow from 24-month-old mice followed by (**c**) ST-HSC and (**d**) MPP. Results were obtained from WT (*n*=6) and *Casp2*^−/−^ (*n*=8) mice at 24 months. Data represented as mean±S.E.M. *P*-values are indicated as either non-significant (ns) or *P<0.05. (**e**) LTC-IC assay. Representative image of colonies obtained from 5 weeks of culture of BM cells from WT and *Casp2*^−/−^ mice. Scale bar=50 *μ*M. (**f**) Quantitation of colonies obtained from LTC-IC assay for WT (*n*=4) and *Casp2*^−/−^ (*n*=4) mice at 24 months. Data represented as mean±S.E.M. *P<0.05

**Figure 2:**
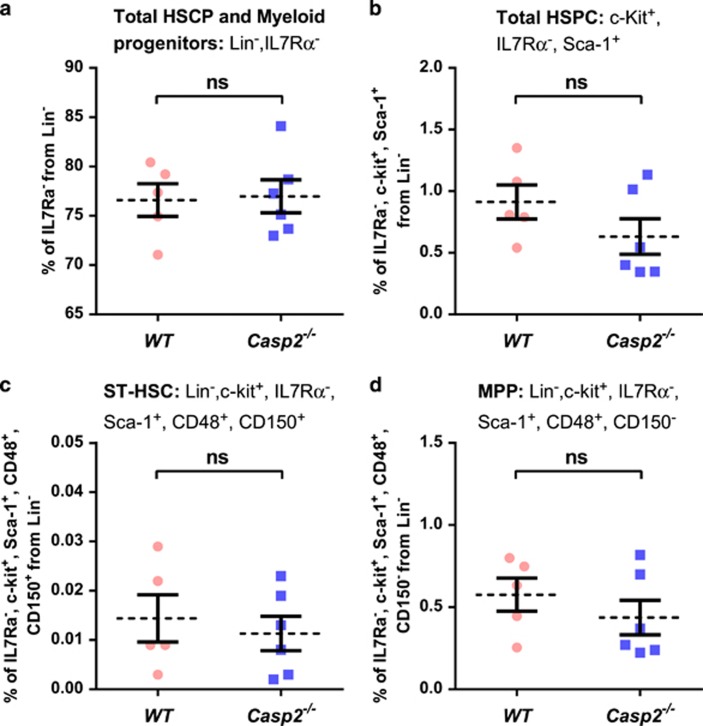
Functional characterization of HSPC in young *Casp2*^−/−^ mice. (**a**) The percentage of total HSPC and myeloid progenitor cells (Lin^−^, IL7R*α*^−^) from BM using flow cytometry. Results were obtained from young WT (*n*=5) and young *Casp2*^−/−^ (*n*=6) mice. Data represented as mean±S.E.M. *P*-values were non-significant (ns). (**b**) The percentage of total HSPCs from c-Kit^+^, Lin^−^ population in the BM of young mice followed by (**c**) ST-HSC and (**d**) MPP. Results were obtained from young WT (*n*=5) and young *Casp2*^−/−^ (*n*=6) mice. Data represented as mean±S.E.M. *P*-values are ns

To test the impact of caspase-2 loss in haematopoiesis with ageing, primary BM cells were isolated from young (4–6 weeks) and aged (18–24 months) *Casp2*^−/−^ mice^25^ and assessed for HSPC fractions by flow cytometry^26, 27^ and for long-term culture-initiating cell (LTC-IC), BM-derived colony-forming unit granulocyte–macrophage (CFU-GM) and serial replating assays. Though total bone marrow morphology was not substantially different between aged WT and *Casp2*^−/−^ mice (Supplementary Figure S1), BM cells from aged *Casp2*^−/−^ mice had significantly higher HSPC and myeloid progenitors (defined by Lin^−^, ILR7*α*^−^) compared with aged WT mice (Figure 1a and b). Similarly, short-term HSC (ST-HSC) (defined by Lin^−^, c-Kit^+^, ILR7*α*^−^, Sca-1^+^, CD48^+^, and CD150^+^) and mutipotent progenitor (MPP) (defined by Lin^−^, c-Kit^+^, ILR7*α*^−^, Sca-1^+^, CD48^+^, CD150^−^) fractions were significantly higher in aged *Casp2*^−/−^ BM cells compared with the aged WT BM cells (Figure 1c and d). In contrast, there were no differences in these BM cell fractions in young WT compared with young *Casp2*^−/−^ mice (Figure 2a–d). These results were further substantiated by LTC-IC and serial replating assays to assess HSPC self-renewal and differentiation capacity.

**Figure 3:**
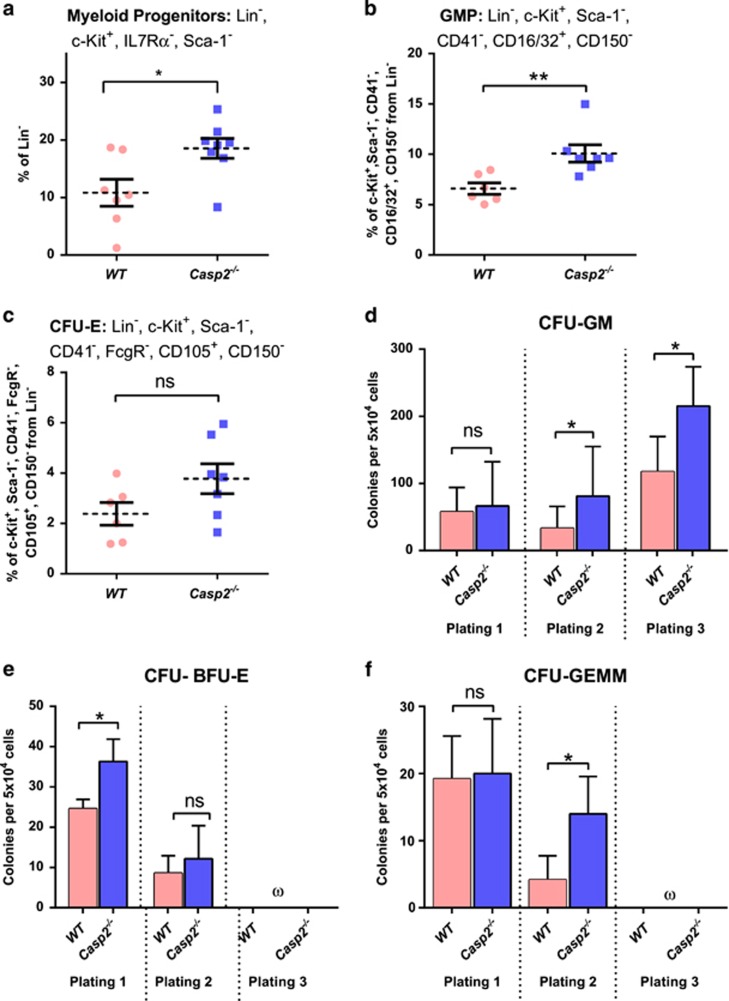
Characterization of myeloid progenitor cells in *Casp2*^−/−^ mice. (**a**) The myeloid progenitor cells from ckit^+^, sca1^−^, Lin^−^, IL7R*α*^−^ from bone marrow quantitated by flow cytometry. Results were obtained from WT (*n*=7) and *Casp2*^−/−^ (*n*=8) mice at 24 months. Data represented as mean±S.E.M. *P*-values are indicated with *P<0.05. (**b**) The percentage of GMP and (**c**) CFU-E in the bone marrow using flow cytometry. Results were obtained from WT (*n*=6) and *Casp2*^−/−^ (*n*=7) mice at 24 months. Data represented as mean±S.E.M. *P*-values are indicated as either non-significant (ns) or **P<0.01. (**d**) Quantitation of myelo-erythroid progenitor self-renewal from aged bone marrow cells (24 months) using serial colony-forming assay. Colonies scored for CFU-GM obtained from WT (*n*=5) and *Casp2*^−/−^(*n*=6) mice. Data represented as mean±S.E.M. *P*-values are indicated as either ns or *P<0.05. (**e** and **f**) Colonies scored for CFU-BFU-E (**e**) and CFU-GEMM **(f**) obtained from WT (*n*=5) and *Casp2*^−/−^(*n*=6) mice. Data represented as mean±S.E.M. *P-*values are indicated as either ns or *P<0.05. The symbol ‘ω' denotes that no colonies were formed

**Figure 4:**
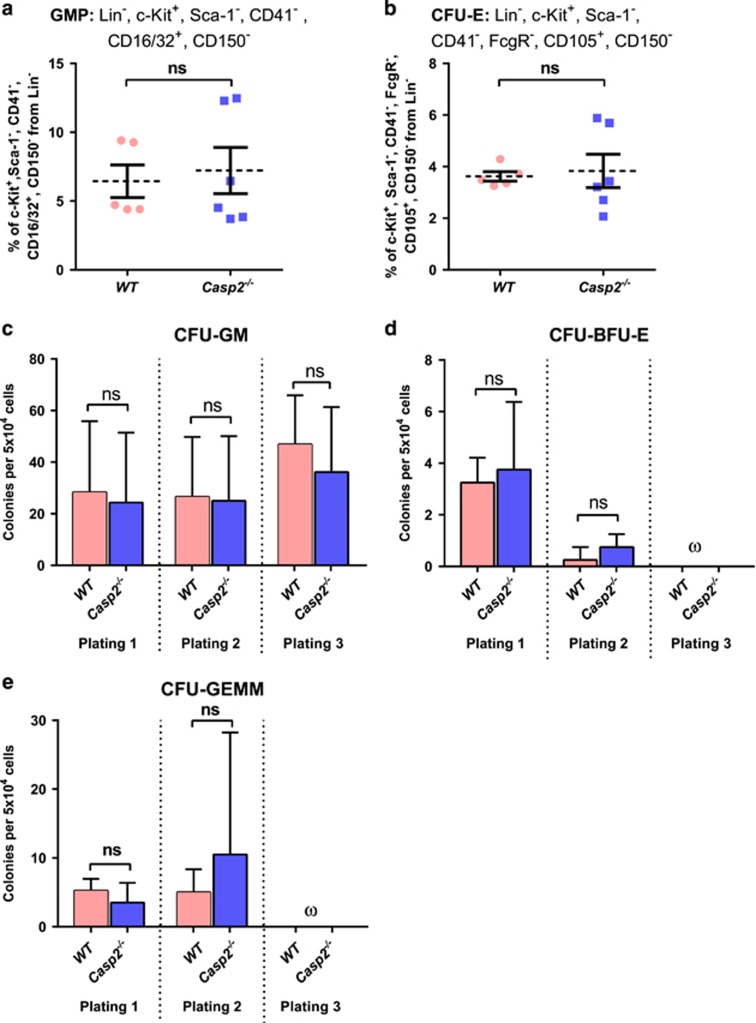
Characterization of myeloid progenitor cells in young *Casp2*^−/−^ mice. The percentage of (**a**) GMP and (**b**) CFU-E in the BM quantitated by flow cytometry. Results were obtained from WT (*n*=5) and *Casp2*^−/−^(*n*=6) mice. Data represented as mean±S.E.M. *P*-values are indicated as non-significant (ns). (**c–e**) Quantitation of myelo-erythroid progenitor self-renewal from BM using serial colony-forming assay. Colonies scored for CFU-GM (**c**), CFU-BFU-E (**d**) and CFU-GEMM (**e**) obtained from WT (*n*=5) and *Casp2*^−/−^(*n*=6) mice. Data represented as mean±S.E.M. *P*-values are indicated as ns. The symbol ‘ω' denotes that no colonies were formed

Assay of total BM from aged *Casp-2*^−/−^ mice was consistent with increased LTC-IC compared with aged WT BM cells (Figure 1e and f). Similarly, serial replating assays of BM mononuclear cells demonstrated a significant increase in burst-forming unit erythroid cells (BFU-E) at first plating for aged *Casp2*^−/−^ compared with aged WT BM cells. In contrast, CFU-GM and CFU granulocyte, erythrocyte, macrophage and megakaryocyte (CFU-GEMM) frequency did not differ at first plating, but these myeloid progenitors displayed increased replating frequency upon secondary and tertiary plating (Figure 3d–f). Colony numbers from primary, secondary or tertiary plating of BM cells from young WT compared with young *Casp2*^−/−^ cells were not different (Figure 4c–e). These results are consistent with caspase-2 deficiency resulting in expansion and enhanced self-renewal of the primitive HSC and myeloid progenitor compartment with ageing.

HSCs are highly susceptible to acquiring precancerous damage such as aneuploidy, DNA damage and skewed differentiation because of their self-renewal and differentiation capacity.^24^ The acquisition of such damage increases with ageing in murine and human bone marrow,^28^ most probably because of their enhanced self-renewal capacity, even though the regenerative capacity of individual stem cells is reduced under stress (i.e., serial transplantation) compared with young HSCs.^29, 30^ Normally, the danger caused by mutagenic accumulation in ageing HSC is prevented by the activity of tumour suppressor proteins that sense potentially malignant clones and trigger apoptosis or growth arrest.^29^ It is therefore plausible that caspase-2 may have an important cell death function in HSCs to prevent survival of potential mutagenized cells to prevent onset of haematopoietic malignancies.

Though aged *Casp2*^−/−^ mice show apparently normal total blood counts, and do not develop spontaneous age-related tumours,^18^ caspase-2-deficiency strongly enhances tumour development in various mouse models.^9, 10, 11, 12, 13, 14^ This indicates that loss of caspase-2 on its own may not be sufficient to induce tumourigenesis, but increases tumour susceptibility in response to cellular stress (i.e. replicative or oncogenic stress). A recent clinical study established that clonal haematopoiesis, with somatic mutations in genes involved in myeloid malignancies, is increasingly common as people age.^31^ Importantly, these individuals harbouring somatic mutations in myeloid genes had normal blood counts at the time of mutation testing, but had increased risk of haematological malignancies during follow-up. Most haematological malignancies arise only after the acquisition of multiple mutagenic events, and the incidence of these haematological malignancies increase with ageing. Gene expression data indicate that *CASP2* expression is also lower in AML stem cells compared with normal HSCs, and within an AML cohort, low *CASP2* expression is associated with poor outcome of AML cases (NCI-Cancer Genome Atlas and BloodSpot databases). In addition, *CASP2* expression is higher in normal human HSCPs compared with mature monocytes and granulocytes (BloodSpot database), probably essential to eliminate damaged HSCPs.

As the ageing haematopoietic system is associated with expansion of myeloid progenitors,^29^ we assessed the impact of caspase-2 deficiency on these progenitors in aged mice. Analysis of the BM myeloid lineage, including myeloid progenitors (MPs) (defined by Lin^−^, c-Kit^+^, ILR7*α*^−^, and Sca-1^−^), granulocyte macrophage progenitors (GMPs) (defined by Lin^−^, c-Kit^+^, Sca-1^−^, CD41^−^, CD16/32^+^, and CD 150^−^), megakaryocyte erythrocyte progenitors (MEPs) (defined by c-Kit^+^, Lin^−^, Sca-1^−^, CD41^−^, CD16/32^−^, CD105^−^, and CD150^+^), megakaryocyte progenitors (MkPs) (defined by c-Kit^+^, Lin^−^, Sca-1^−^, CD41^+^, and CD150^+^) and erythroid progenitors (CFU-E) (defined by Lin^−^, c-Kit^+^, Sca-1^−^, CD41^−^, CD16/32^−^, CD105^+^, and CD150^−^), was performed in both young and aged mice. Aged *Casp2*^−/−^ BM cells exhibited significantly increased frequency of myeloid progenitors with a trend towards enhanced GMP populations (Figure 3a and b) compared with aged WT BM cells. Aged *Casp2*^−/−^ BM cells also showed a trend of higher erythroid progenitor populations (Figure 3c) compared with aged WT BM cells. Intriguingly, no differences were seen in these progenitor cell populations from young WT and *Casp2*^−/−^ mice (Figure 4a and b). This result, combined with the clonal assays in Figure 3d–f, suggest that loss of caspase-2 results in a skewing of haematopoiesis towards the myeloid lineage with ageing. These results are consistent with a study demonstrating age-related skewing towards myelopoiesis in adult human BM and further indicate that loss of caspase-2 enhances this phenotype.^30^

**Figure 5:**
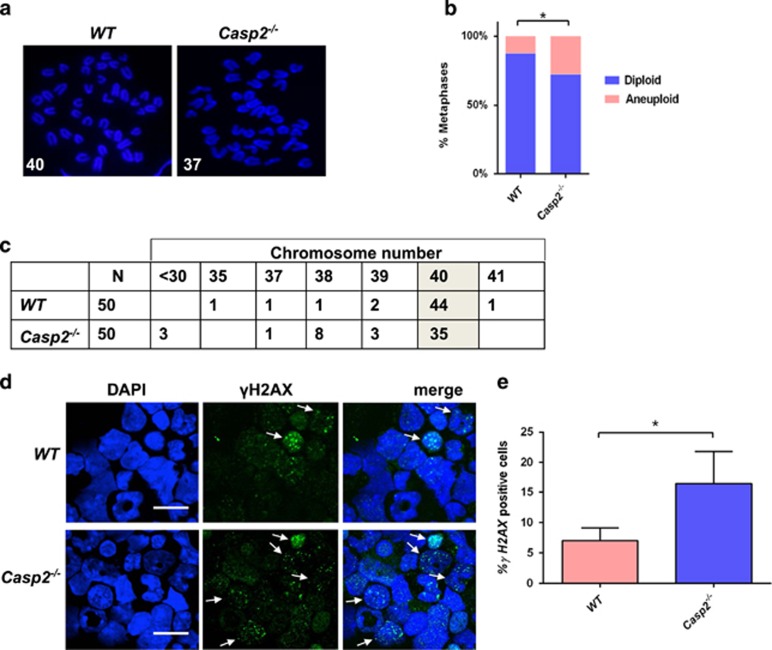
Caspase-2 deficiency enhances aneuploidy and DNA damage in primary BM cells with ageing. (**a**) Representative fluorescent images (magnification of 100 ×) of DAPI-stained metaphase spreads, with chromosome counts indicated, from aged WT (*n*=4) and aged *Casp2*^−/−^ (*n*=4) BM cells. (**b**) Quantitation of metaphases showing frequencies of diploid and aneuploid karyotypes in BM cells from aged WT (*n*=4) and *Casp2*^−/−^ (*n*=4) mice. A total of 50 metaphase spreads were counted per mouse. Data represented as mean±S.E.M. and *P<0.05, *χ*^2^ test. (**c**) Table indicating actual chromosome numbers from aged WT and aged *Casp2*^−/−^ BM cells showing reduced cells with diploid number of chromosomes indicated (shaded) and increased aneuploidy in *Casp2*^−/−^ BM cells. (**d**) Representative fluorescent images (magnification of63 ×) of phospho-histone H2AX (Ser139)-stained BM cells from aged WT (*n*=4) and aged *Casp2*^−/−^ (*n*=4) mice. Arrows highlight cells with multiple *γ*H2AX foci. Scale bar=10 *μ*M. (**e**) Quantitation of phospho-histone H2AX-positive nuclei of BM cells from aged WT (*n*=4) and *Casp2*^−/−^ (*n*=4) mice. Data represented as mean±S.E.M. and *P<0.05

**Figure 6:**
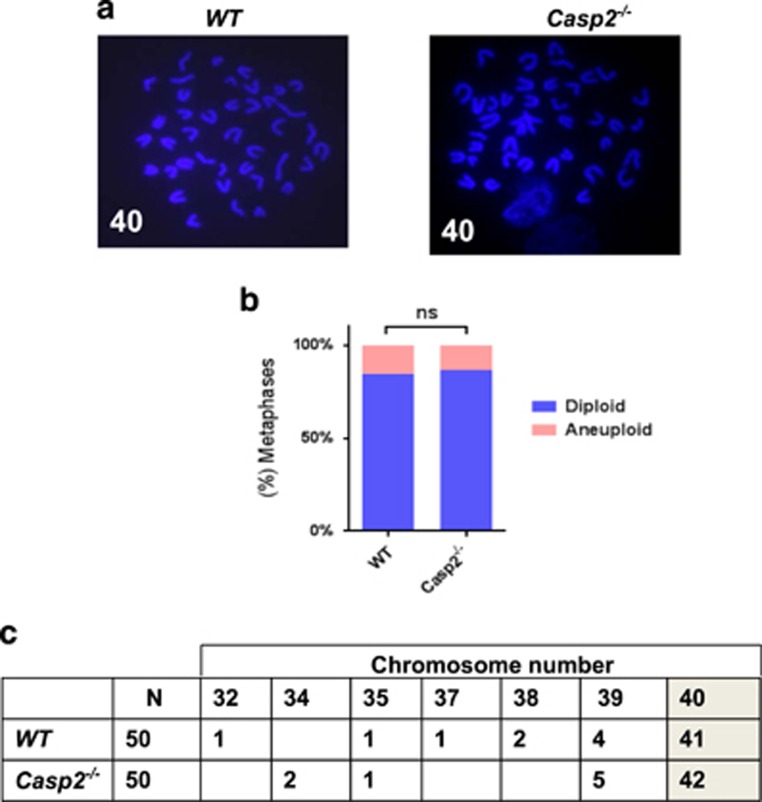
Caspase-2 deficiency does not enhance aneuploidy in primary BM cells from young mice. (**a**) Representative fluorescent images (magnification of100 ×) of DAPI-stained metaphase spreads, with chromosome counts indicated, from young WT (*n*=4) and young *Casp2*^−/−^ (*n*=4) BM cells. (**b**) Quantitation of metaphases showing frequencies of diploid and aneuploid karyotypes in BM cells from young WT (*n*=4) and young *Casp2*^−/−^ (*n*=4) mice. A total of 50 metaphase spreads were counted per mouse. *P-*value is indicated as non-significant (ns). (**c**) Table indicating actual chromosome numbers from young WT and young *Casp2*^−/−^ BM cells showing no difference in diploid number of chromosomes (shaded)

As stem cells are susceptible to acquiring mitotic defects with age because of their self-renewal capacity,^23, 24^ we examined whether loss of *caspase-2* exacerbates age-related aneuploidy in BM. Indeed, aged *Casp2*^−/−^ mice, but not young mice, exhibited significantly enhanced aneuploidy in BM compared with WT mice (Figures 5a–c and 6a–c). These results indicate that *Casp2*^−/−^ BM cells are more susceptible to aneuploidy *in vivo* with ageing and also may explain the association of reduced caspase-2 expression with poor survival in AML, as aneuploidy is a negative prognostic indicator.^32, 33^ As ageing is associated with accumulation of DNA damage in HSC,^24, 34^ we assessed this and observed significantly enhanced basal *γ*H2AX foci together with an increased number of *γ*H2AX-positive cells in aged *Casp2*^−/−^ BM compared with aged WT cells (Figure 5d and e). Together, these results suggest that caspase-2 deficiency enhances aneuploidy associated with increased DNA damage in BM with ageing.

## Discussion

Caspase-2 deficiency has previously been linked to enhanced haematopoietic malignancy in both humans and mouse models. In this work we now demonstrate a previously unrecognized role for caspase-2 in maintaining HSC homeostasis with ageing that may play a role in premature ageing and enhanced susceptibility to tumourigenesis associated with *Casp2*^−/−^ animals. We also show for the first time that caspase-2 loss alone is sufficient for accumulation of aneuploid cells in the BM with age. Our previous work has shown that *caspase-2*-deficient tumours, as well as MEFs, have increased tendency to become aneuploid and has suggested that this could be because of reduced cell death in a *caspase-2*-deficient background.^11, 15^ Our recent work with live cell imaging of mouse splenocytes *ex vivo* has demonstrated that caspase-2 is required for deleting mitotically aberrant cells, including aneuploid cells, and that the absence of caspase-2 can promote the long-term survival of such aberrant cells.^34^ Thus, the accumulation of aneuploid cells in the BM of *Casp2*^−/−^ animals with age is consistent with the idea that caspase-2-dependent cell death limits aneuploidy, given BM is the compartment where there is continuous cell division, thus increasing the likelihood of these cells having mitotic aberrations.

In the BM, one of the consequences of ageing is that blood cell composition becomes skewed toward myeloid cells, with increased incidence of myelogenous cancer, whereas the main forms of acute leukemia in children is of the lymphoid lineages.^28, 29, 30, 31^ Our previous studies have demonstrated that aged *Casp2*^−/−^ mice exhibit increased reactive oxygen species (ROS) and reduced stress tolerance that is associated with increased DNA damage.^15, 18, 20^ ROS-related DNA damage is a well-established factor known to compromise HSC renewal and differentiation capacity with ageing.^35^ A robust oxidative stress response, together with accurate repair of DNA damage, are critical for HSC maintenance and protection against functional decline of HSCs during ageing.^29, 35^ The BM compartment comprises a low-oxygenic niche to ensure protection from ROS-induced stress and damage and this protection is lost during the ageing process.^36^ ROS is also known to play a critical role in lineage decision of myeloid progenitors and can promote differentiation into GMP.^37^ Therefore, it is possible that the increased ROS in *Casp2*^−/−^ mice contributes to the observed age-related DNA damage and aneuploidy, as well as the enhanced myeloid skewing with age.

As the precise mechanisms underlying haematopoietic ageing remain poorly understood, the *Casp2*^−/−^ mice may provide a useful tool to further develop this hypothesis. Additional studies will be important to determine the molecular pathway by which caspase-2 regulates HSC and progenitor genomic stability and differentiation, and to assess any increase in susceptibility of aged *Casp2*^−/−^ mice to leukaemia. Such knowledge will provide insight into the causative factors associated with increased incidence of haematological malignancies with ageing.

## Materials and methods

### Mice

*Caspase-2* knockout (*Casp2*^−/−^) mice have been described previously.^18, 25^ These mice have been backcrossed to a C57BL/6 background for at least 20 generations. Animals were maintained in specific pathogen-free conditions in a 12 h/12 h light/dark cycle and all animal breeding and studies were approved by the SA Pathology/Central Northern Adelaide Health Services Animal Ethics Committee.

### Flow cytometry analysis

BM cells were stained and analysed according to previously described protocols.^26, 27^ For the stem cell and lymphoid progenitor panel, 8 × 10^5^ of BM cells were stained with the following antibodies: FITC anti-mouse CD48 (BioLegend, San Diego, CA, USA), Anti-mouse CD135 (Flt3) (eBioscience, San Diego, CA, USA), APC anti-mouse CD150 (SLAM) (BioLegend), Anti-mouse CD117 (c-Kit) APC-eFluor (eBioscience), Pacific Blue anti-mouse Ly-6A/E (Sca-1) (BioLegend), Biotin anti-mouse CD127 (IL-7R*α*) (BioLegend), StrepAvidin-PECy7 (BioLegend) and lineage antibodies (CD3e-PECy5 (eBioscience), B220-PECy5 (BioLegend), Gr1-PECy5 (BioLegend), Mac1-PECy5 (BioLegend) and TER-119-PECy5 (BioLegend)). The gating strategy for stem and lymphoid progenitor cell analysis is shown in Supplementary Figure S2.

For the myeloid progenitor panel, 8 × 10^5^ BM cells were stained with the following antibodies: FITC rat anti-mouse CD41 (BD Pharmingen, San Jose, CA, USA), PE rat anti-mouse CD16/32 (BD Pharmingen), APC anti-mouse CD150 (SLAM) (BioLegend), Anti-mouse CD117 (c-Kit) APC-eFluor (eBioscience), Pacific Blue anti-mouse Ly-6A/E (Sca-1) (BioLegend), PE/Cy7 anti-mouse CD105 (BioLegend) and lineage antibodies (PE/Cy5 anti-mouse CD3*ɛ* (eBioscience), PE/Cy5 anti-mouse CD11b (BioLegend), PE/Cy5 anti-mouse CD45R/B220 (BioLegend), PE/Cy5 anti-mouse Ly-6G/Ly-6C (Gr-1) (BioLegend) and PE/Cy5 anti-mouse TER-119 (BioLegend)). Samples were run on Fortressa Flow Cytometer (BD Biosciences, San Jose, CA, USA) and data analysis was performed using FCS Express Flow Cytometry Research Edition Version 4 (DeNovo Software, Glendale, CA, USA). The gating strategy for myeloid progenitor cell analysis is shown in Supplementary Figure S3.

### CFU assay

Methylcellulose colony-forming assays were performed using MethoCult GF M3434 (Stem Cell Technologies, Vancouver, BC, Canada) according to the manufacturer's protocol.^38^ Briefly, 1 × 10^4^ BM cells were seeded in triplicate. CFU-GM and spontaneous BFU-E colonies, >50 cells in size, were counted at day 7 post seeding. Representative photographs for colony morphology were taken using an Olympus CK2 microscope (Olympus Corporation, Japan, Tokyo) and DP11 camera system at a magnification of 100 ×. For serial replating assays, cells from the triplicate day 7 post seeding were harvested and washed 3 times in IMDM media to wash away the MethoCult. Again, 1 × 10^4^ BM cells were seeded in triplicate. At day 7 post seeding, colonies were scored and the same procedures were repeated.

### LTC-IC assay

LTC-IC assay was carried out as described previously.^39^ BM cells were plated in duplicate on AFT024 feeder cell layers (irradiated with 20 Gy) established in 24-well plates in MyeloCult M5300 (Stem Cell Technologies) freshly supplemented with 10^−6^ M hydrocortisone. Cultures were maintained for 5 weeks at 37 °C with weekly half-media changes before seeding the harvested cells for CFU assays. Representative colony images were taken using a stereomicroscope (Nikon, Tokyo, Japan).

### Cytogenetic and DNA damage analysis

Preparation of chromosome spreads and DNA damage analysis was carried out as previously described.^11, 15^ Chromosome spreads were prepared by adding colcemid (20 ng/ml) 4 h before harvesting cells by trypsinization, hypotonic treatment (0.075 M KCl) and fixation in fresh ice-cold Carnoy's fixative (methanol/glacial acetic acid at 3 : 1) for 10 min at 37 °C. Cells were centrifuged at 1000 r.p.m., washed three times in Carnoy's fixative and dropped onto wet glass slides, air dried and then placed in a 60 °C oven overnight. Cells were stained with DAPI and chromosomes quantitated by epifluorescence microscopy (model BX51; Olympus) and camera (UCMAD3/CVM300, Olympus). Cells were visualized under 40 × or 100 × ULAPO objective lens with NA=1.5. Images were processed using Olysia BioReport Software (Olympus) and manually merged using Adobe Photoshop 6.0 software (Adobe Systems Inc., San Jose, CA, USA).

For DNA damage analysis, primary BM cells (1 × 10^5^) were fixed in 4% w/v paraformaldehyde for 15 min at 4 °C, permeabilized with 0.25% v/v Triton X-100/PBS for 10 min at RT and then blocked in 1% w/v BSA/PBS for 1 h at RT. Cells were incubated with anti-phospho-histone H2AX (Ser139) primary antibody (Cell Signaling Technology, Danvers, MA, USA) diluted in blocking buffer, overnight at 4 °C. Following 3 washes in blocking buffer, cells were incubated with donkey anti-rabbit Alexa Fluor 488 secondary antibody (Life Technologies, Carlsbad, CA, USA) diluted 1 : 1000 in blocking buffer for 1 h at RT. Following antibody incubations and washes in PBS, cells were counterstained with 2 *μ*g/ml DAPI for 5 min and mounted with ProLong Gold Antifade Reagent (Thermo Fisher Scientific, Waltham, MA, USA). At least 100 cells were scored for each sample. Fluorescent images were captured using a LSM 800 confocal laser scanning microscope (Zeiss, Oberkochen, Germany).

### Giemsa staining

BM cell cytospins were stained using Aerospray Hematology Pro (EliTech Group, Puteaux, France) by the Department of Clinical Pathology, SA Pathology (Adelaide, SA, Australia) and analysed by an expert haematologist. The images were captured using an epifluorescence microscopy (model BX51; Olympus) and camera (UCMAD3/CVM300, Olympus).

### Statistical analysis of data

Statistical analysis was carried out in GraphPad Prism, Version 6.05 (GraphPad, GraphPad Inc., La Jolla, CA, USA). Student's *t*-test was used for statistical significance analysis to determine *P*-values between each genotype, unless otherwise stated. Data are expressed as mean±S.E.M. *P*<0.05 was considered significant.
