# Supplementary material for: Impaired haematopoietic stem cell differentiation and enhanced skewing towards myeloid progenitors in aged caspase-2-deficient mice
Source: Cell Death Dis. 2016 Dec 1;7(12):e2509–. doi: 10.1038/cddis.2016.406 (PMC5260989; doi:10.1038/cddis.2016.406)
Supplement: Supplementary Figure Legends [file cddis2016406x1.docx]

## Supplementary Information

**Impaired haematopoietic stem cell differentiation and enhanced skewing towards myeloid progenitors in aged caspase-2-deficient mice**

Swati Dawar, Nur Hezrin Shahrin, Nikolina Sladojevic, Richard J. D’Andrea, Loretta Dorstyn, Devendra K. Hiwase, Sharad Kumar

**Supplementary Figure Legends**

**Supplementary Figure S1. Representative BM morphology images of two aged WT and *Casp2^-/-^* mice.**

The images were captured at **(a)** 4X, **(b)** 20X and **(c)** 100X respectively. There was no substantial difference in cellularity and morphology of the haematopoietic cells.

**Supplementary Figure S2. Gating strategy for the analysis of stem and lymphoid progenitor cells by flow cytometry.**

**(a)** The live cell populations were determined by FVS700 negative staining. **(b)** Cell debris was excluded from the analysis based on forward and side scatter. **(c)** Plot indicating selection of Lin^-^ and c-Kit^+^ cells. **(d)** Cells were gated for either IL7Rα^+^ positive or IL7Rα^-^ for selection of common lymphoid progenitor (CLP) or HSC respectively. **(e)** Plot indicating the total HSPC population selected from Sca-1^+^ cells. **(f)** Plot showing gating of cells for LT-HSC, ST-HSC and MPP. The indicated percentages of the lymphoid progenitor cell populations are representative values of cells isolated from WT BM cells and were similar to cell populations isolated from *Casp2^-/-^* BM.

**Supplementary Figure S3**. **Diagrammatic representation of gating strategy for analysis of myeloid progenitor cells with ageing.**

**(a)** The live cell population was determined by FVS700 negative staining. **(b)** Cell debris was excluded from the analysis based on forward and side scatter. **(c)** Plots showing selection for c-Kit^+^, Lin^-^ cells. **(d)** Plot showing Sca-1^-^ cell population used for the myeloid progenitor cell selection. **(e-g)** The last three plots demonstrate the purified myelo-erythroid progenitor subsets including **(e)** MkP (CD41^+^) (Megakaryocytic progenitor), **(f)** GMP (Granulocyte/macrophage progenitor), **(g)** CFU-E and Pre CFU-E, (Colony Forming Unit - Erythrocyte), Pre MegE (Megakaryocyte/Erythrocyte), and Pre GM (Granulocyte/Macrophage) cells. The indicated percentages of the myeloid progenitor cell populations are representative values of cells isolated from WT BM cells and were similar to cell populations isolated from *Casp2^-/-^* BM.
