# Supplementary figures and images for: Impaired haematopoietic stem cell differentiation and enhanced skewing towards myeloid progenitors in aged caspase-2-deficient mice
Source: Cell Death Dis. 2016 Dec 1;7(12):e2509–. doi: 10.1038/cddis.2016.406 (PMC5260989; doi:10.1038/cddis.2016.406)

Supplementary Figure 1

**Aged WT**

**Aged *Casp2*<sup>-/-</sup>**

1

2

1

2

**a**

4X

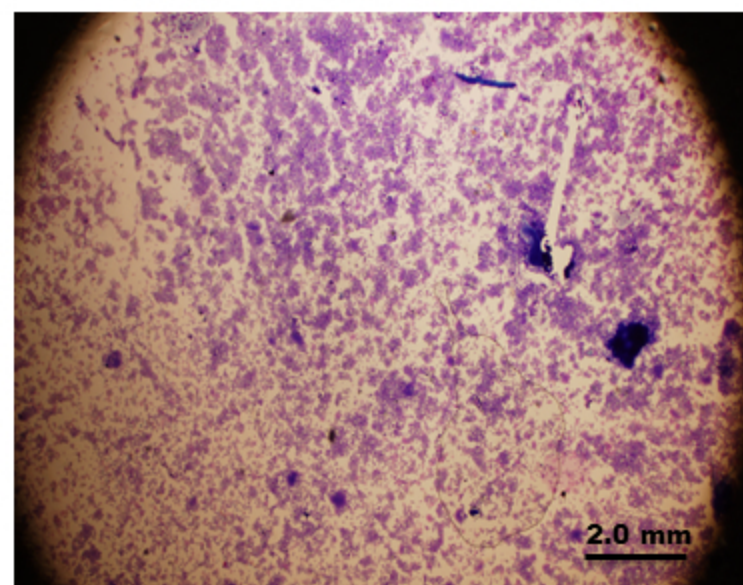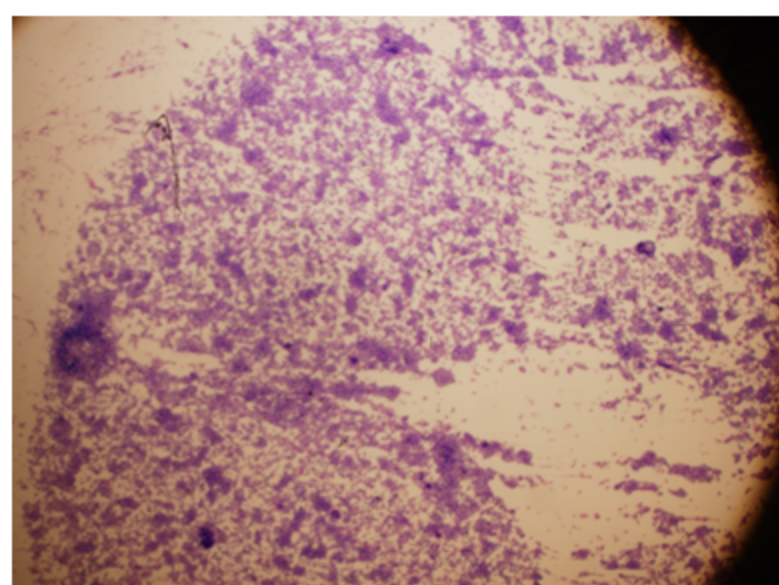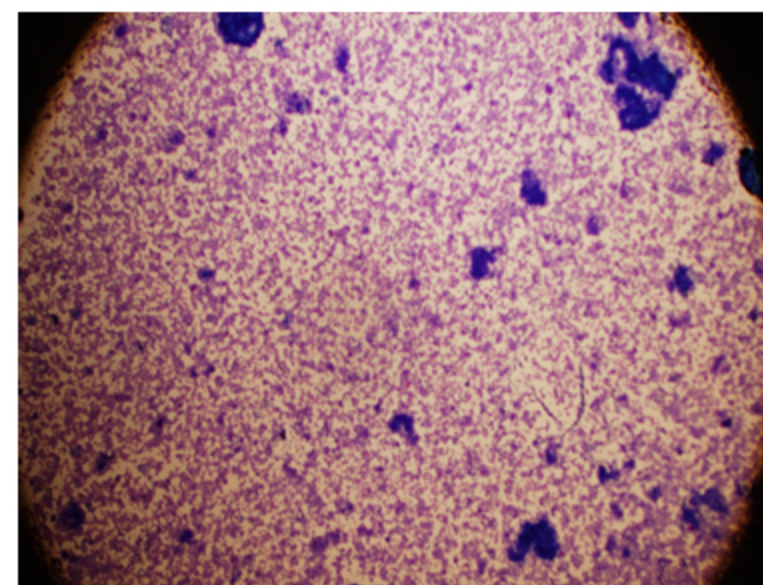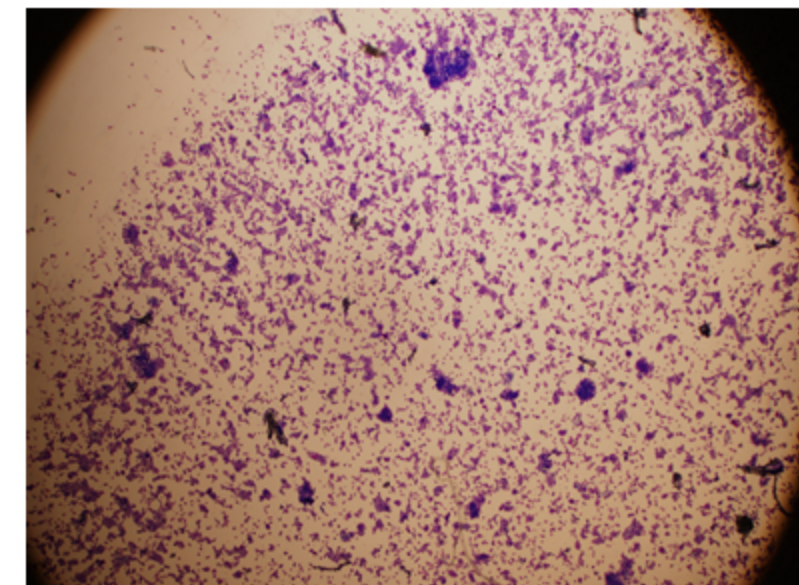

**b**

20X

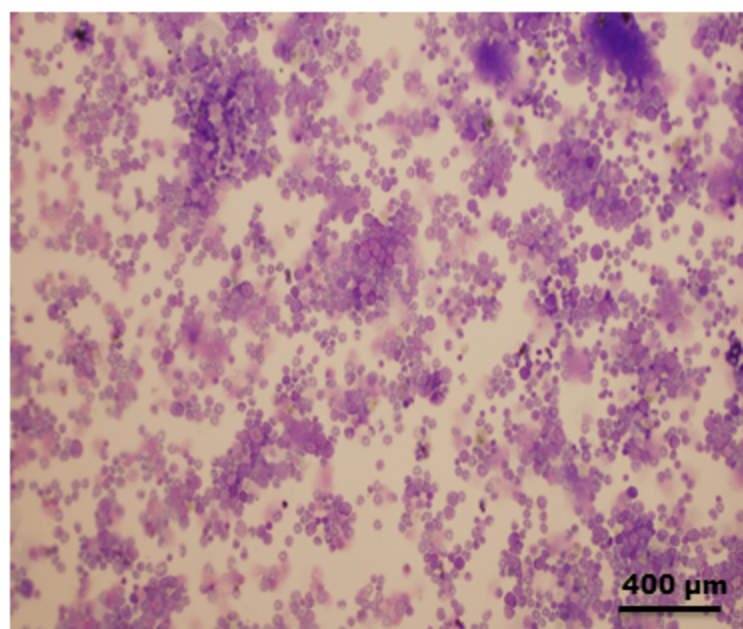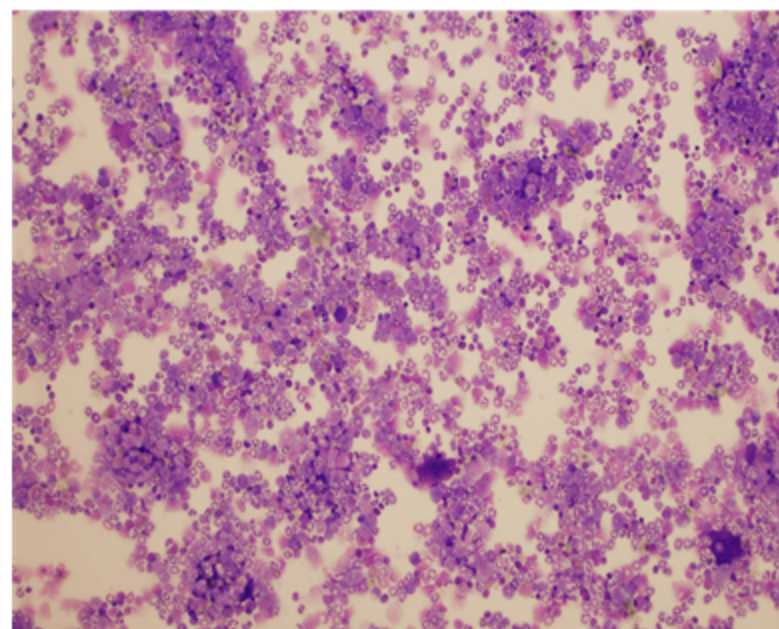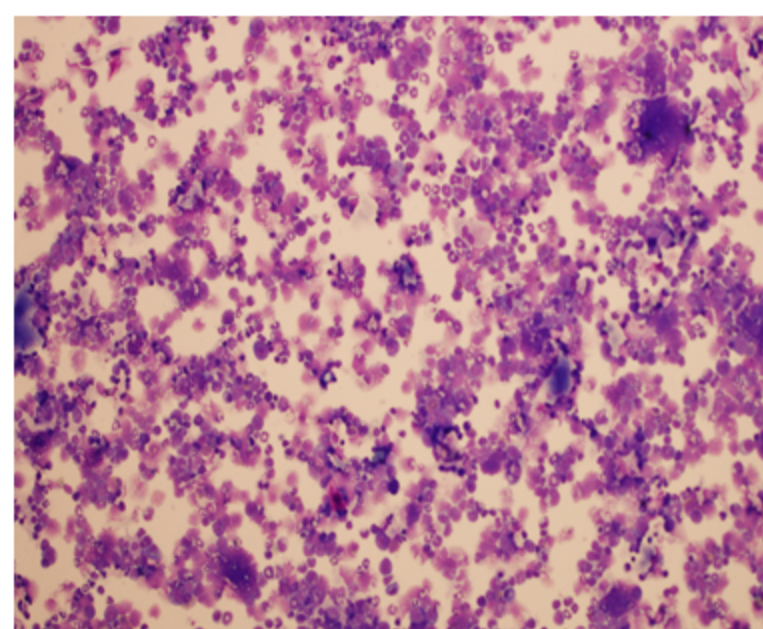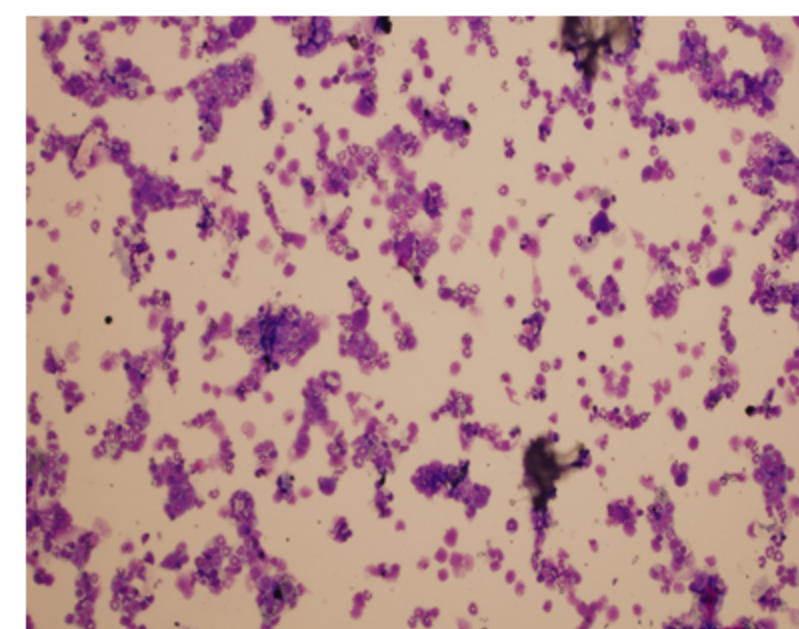

**c**

100X

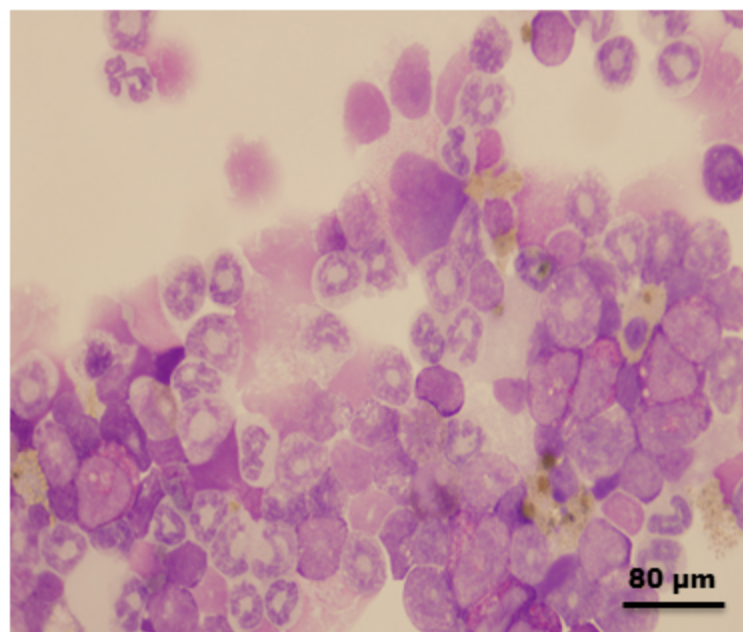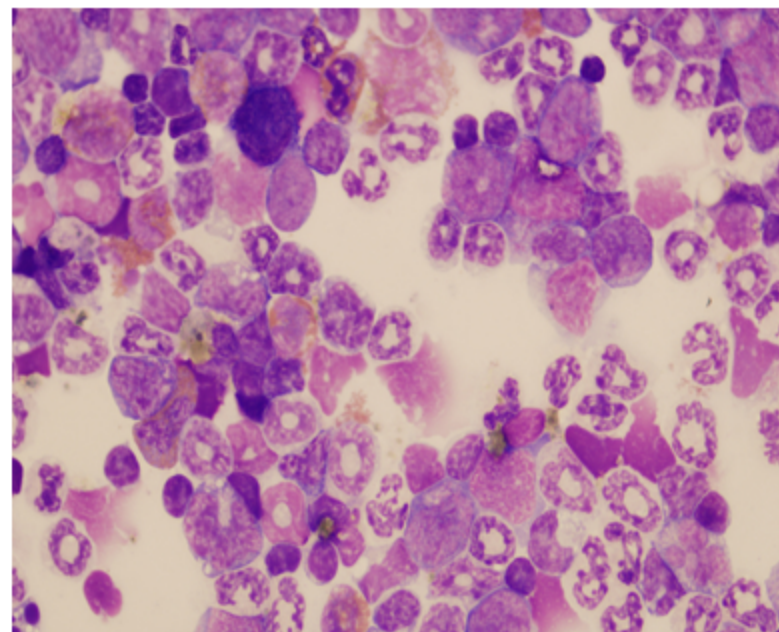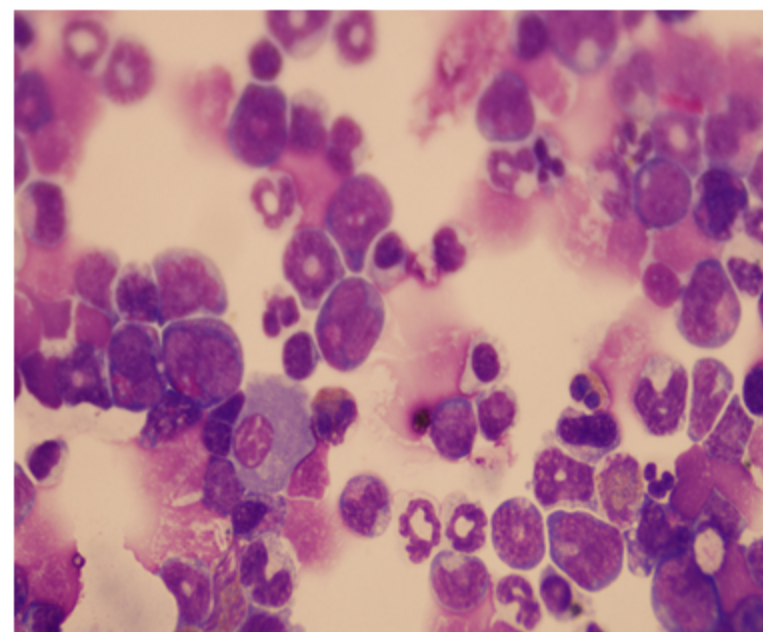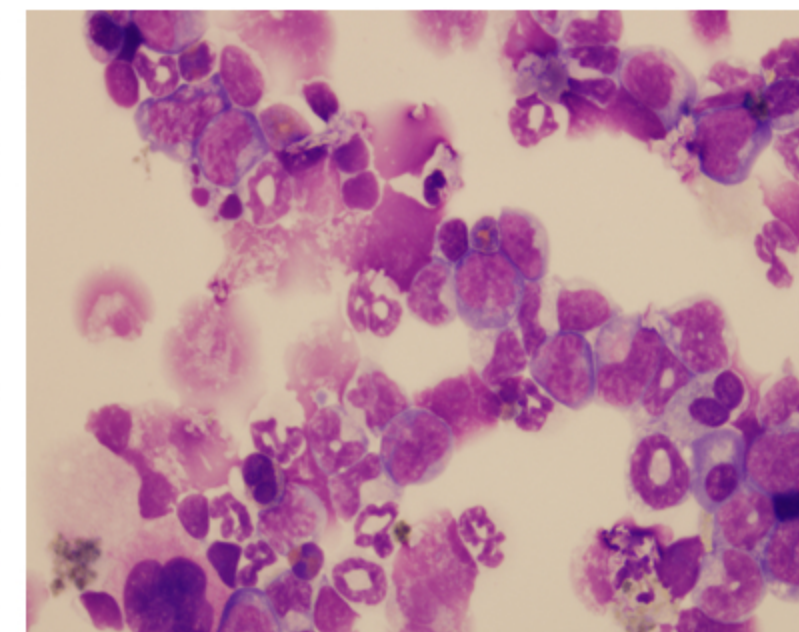

Supplement: Supplementary Figure S1 [file cddis2016406x2.pdf]

Supplementary Figure S2

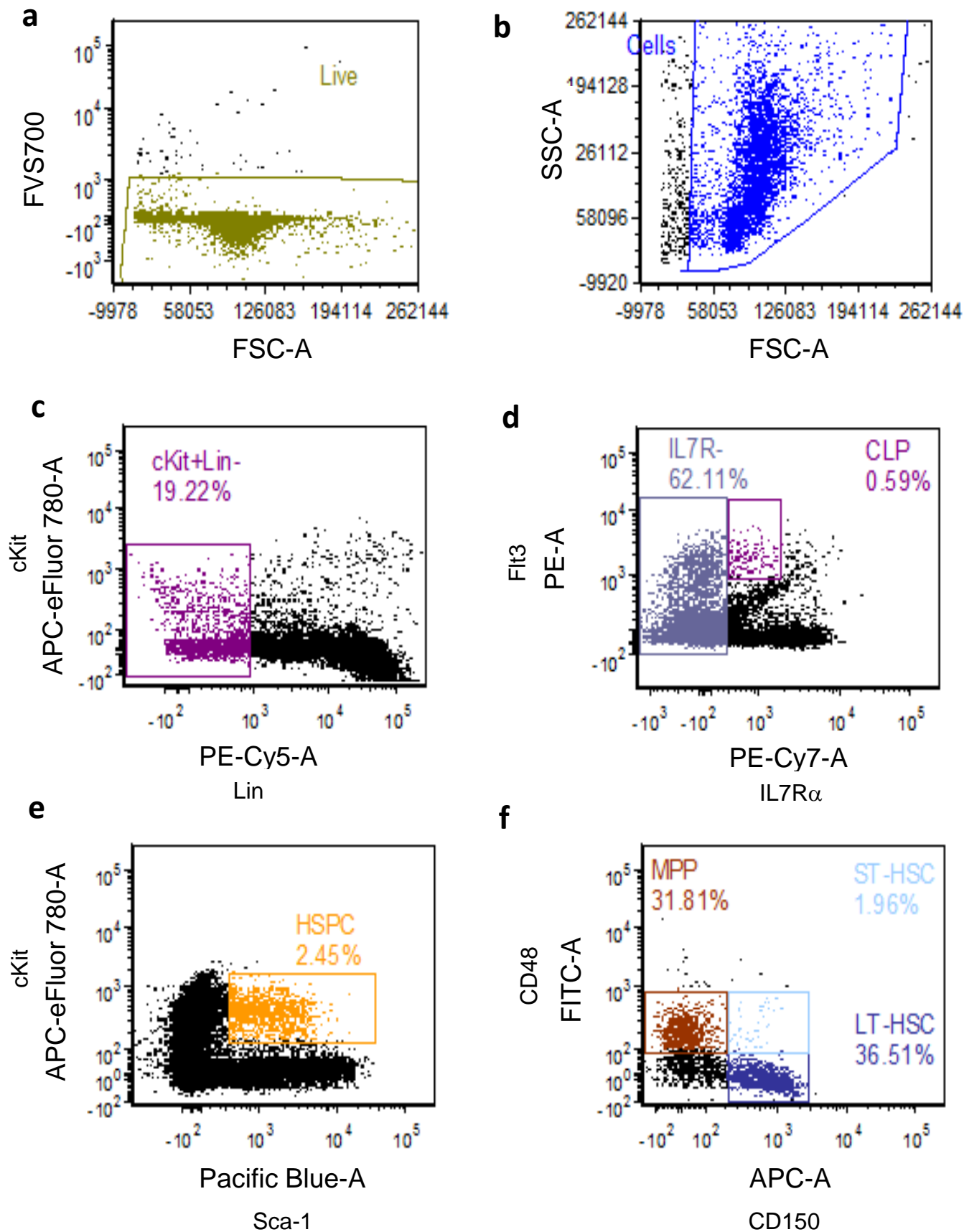

Supplement: Supplementary Figure S2 [file cddis2016406x3.pdf]

# Supplementary Figure S3

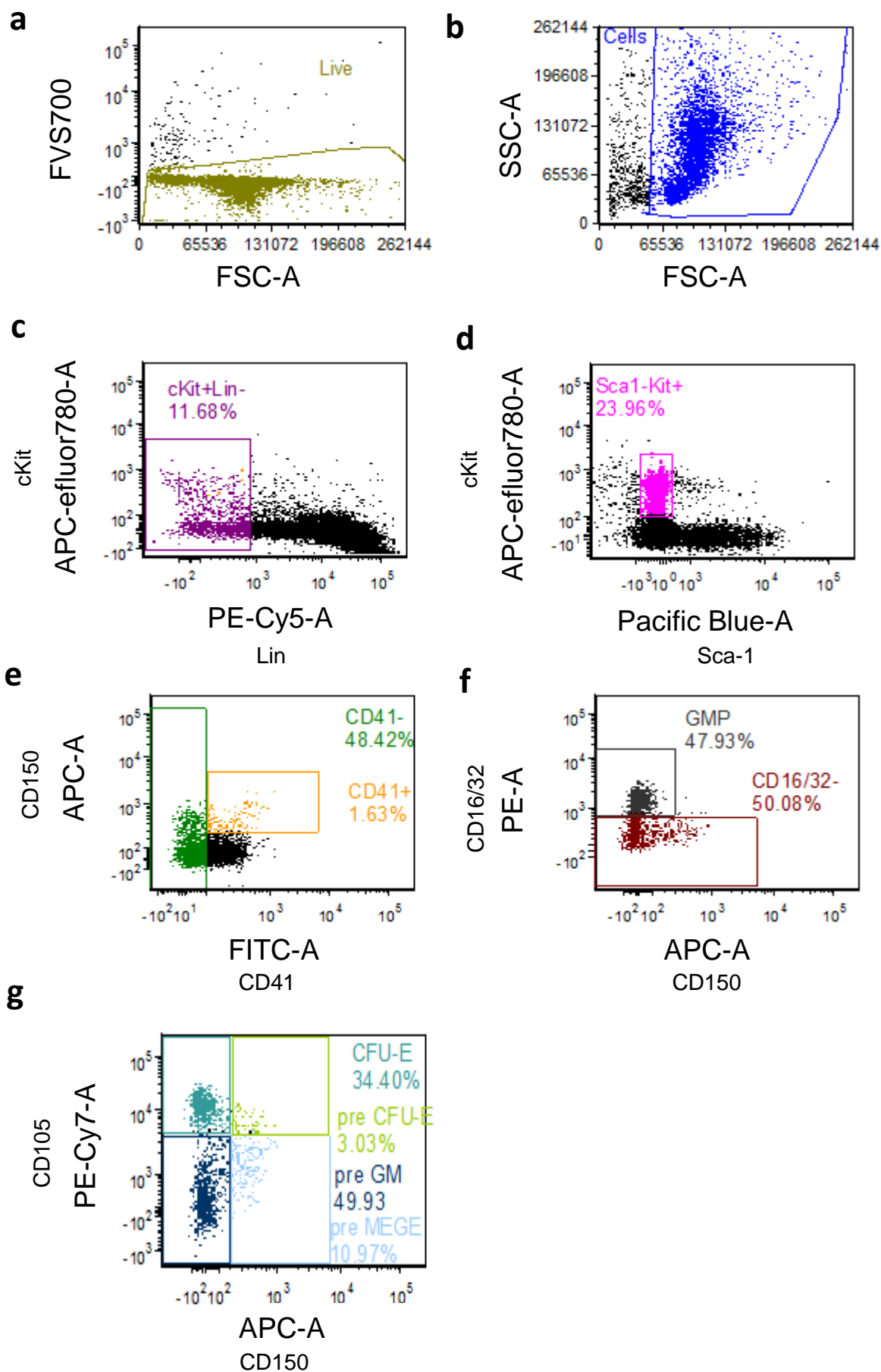

Supplement: Supplementary Figure S3 [file cddis2016406x4.pdf]
